# Effects of patterned peripheral nerve stimulation on soleus spinal motor neuron excitability

**DOI:** 10.1371/journal.pone.0192471

**Published:** 2018-02-16

**Authors:** Samuel Jimenez, Laura Mordillo-Mateos, Michele Dileone, Michela Campolo, Carmen Carrasco-Lopez, Fabricia Moitinho-Ferreira, Tomas Gallego-Izquierdo, Hartwig R. Siebner, Josep Valls-Solé, Juan Aguilar, Antonio Oliviero

**Affiliations:** 1 FENNSI Group, Hospital Nacional de Parapléjicos, SESCAM, Toledo, Spain; 2 Physiotherapy Department, Centro Superior de Estudios Universitarios La Salle, Universidad Autónoma de Madrid, Madrid, Spain; 3 Physiotherapy Department, Alcalá de Henares University, Alcalá de Henares Spain; 4 CINAC, HM Puerta del Sur, Hospitales de Madrid, Móstoles, Spain; 5 EMG and Motor Control Section, Neurology Department, Hospital Clinic, University of Barcelona, Barcelona, Spain; 6 Sarah Network of Rehabilitation Hospitals, Salvador de Bahia, Brazil; 7 Danish Research Centre for Magnetic Resonance, Centre for Functional and Diagnostic Imaging and Research, Copenhagen University Hospital, Hvidovre, Hvidovre, Denmark; 8 Department of Neurology, Copenhagen University Hospital Bispebjerg, Copenhagen, Denmark; 9 Experimental Neurophysiology Group, Hospital Nacional de Parapléjicos, SESCAM, Toledo, Spain; University of Ottawa, CANADA

## Abstract

Spinal plasticity is thought to contribute to sensorimotor recovery of limb function in several neurological disorders and can be experimentally induced in animals and humans using different stimulation protocols. In healthy individuals, electrical continuous Theta Burst Stimulation (TBS) of the median nerve has been shown to change spinal motoneuron excitability in the cervical spinal cord as indexed by a change in mean H-reflex amplitude in the flexor carpi radialis muscle. It is unknown whether continuous TBS of a peripheral nerve can also shift motoneuron excitability in the lower limb. In 26 healthy subjects, we examined the effects of electrical TBS given to the tibial nerve in the popliteal fossa on the excitability of lumbar spinal motoneurons as measured by H-reflex amplitude of the soleus muscle evoked by tibial nerve stimulation. Continuous TBS was given at 110% of H-reflex threshold intensity and compared to non-patterned regular electrical stimulation at 15 Hz. To disclose any pain-induced effects, we also tested the effects of TBS at individual sensory threshold. Moreover, in a subgroup of subjects we evaluated paired-pulse inhibition of H-reflex. Continuous TBS at 110% of H-reflex threshold intensity induced a short-term reduction of H-reflex amplitude. The other stimulation conditions produced no after effects. Paired-pulse H-reflex inhibition was not modulated by continuous TBS or non-patterned repetitive stimulation at 15 Hz. An effect of pain on the results obtained was discarded, since non-patterned 15 Hz stimulation at 110% HT led to pain scores similar to those induced by EcTBS at 110% HT, but was not able to induce any modulation of the H reflex amplitude. Together, the results provide first time evidence that peripheral continuous TBS induces a short-lasting change in the excitability of spinal motoneurons in lower limb circuitries. Future studies need to investigate how the TBS protocol can be optimized to produce a larger and longer effect on spinal cord physiology and whether this might be a useful intervention in patients with excessive excitability of the spinal motorneurons.

## Introduction

Spinal plasticity can be triggered in animal and human spinal cord using different experimental protocols [1,2] For instance, spinal plasticity can be induced by behavioural manipulation [3], or by repetitive electrical stimulation of the ventral horn of the spinal cord *in vitro*[4].

In humans, the monosegmental and monosynaptic H-reflex can be recorded in a few muscles to estimate trans-synaptic excitability of the spinal motorneurons through peripheral afferents [5]. H-reflex measurements have been widely used to assess the impact of non-invasive stimulation techniques on the excitability of spinal motorneurones. Some stimulation techniques directly target the spinal cord: for example transcutaneous spinal Direct Current Stimulation (tsDCS) modulates H-reflex [6,7]. Other stimulation techniques target the intraspinal circuitry indirectly via electrical stimulation of peripheral nerves or transcranial magnetic stimulation (TMS) of the fast-conducting descending motor pathways [8,9].

Some of these techniques use trains of stimuli delivered in a uniform way: electrical tetanic peripheral nerve stimulation successfully increased H-reflex amplitude [10]. On the other hand, patterned stimulation protocols allowed obtaining similar effects on spinal and cortical excitability [11–15]. A form of patterned stimulation known to produce robust effects in the central nervous system is the theta burst stimulation (TBS). Depending on whether TBS is given intermittently [i.e. intermittent TBS (iTBS)] or continuously [continuous TBS (cTBS)], TBS tends to induce LTP-like or LTD-like effects, respectively [13,16].

The idea of using TBS to induce plasticity originated from the observation in rat hippocampus that short high-frequency bursts that resemble in vivo hippocampal activity patterns could induce a stable potentiation of postsynaptic responses when bursts were delivered at 4–7 Hz (the theta range) [17–20]. However, the mechanisms of TBS in humans are largely unknown. Recently, Yeh and coworkers [21] found that the application of a repetitive electrical stimulation protocol adapted from magnetic cTBS to the median nerve modulated spinal cord excitability evaluated by H-reflex recordings. In particular, the authors found that the effects on H reflex amplitude were dependent on the stimulation intensity used: H/M ratio was facilitated when an intensity equal to 110% H-reflex threshold was used, while it was reduced when using an intensity equal to 90% H-reflex threshold. The effects were long-lasting and correlated to the duration of the stimulation itself (30 minutes with stimulation lasting 40 s and 45 minutes with stimulation lasting 80 s).

Prompted by the finding that electrical cTBS of the median nerve can modulate the size of the H-reflex in the upper limb [21], we aimed to explore the properties of neural plasticity of spinal circuitry in lower limbs muscles by applying peripheral nerve cTBS and evaluating soleus H-reflex amplitude in normal subjects. The spinal networks engaged in the control of reflex circuits of the lower limbs are functionally different from those controlling upper limbs and, therefore, the findings obtained in the upper limb may not apply to the reflex responses of the lower limb [22–24]. Therefore, lower and upper limbs could have a different propensity to plasticity phenomena.

To study a dose-effect curve of the stimulation protocol we performed two different experimental sessions with the same protocol (i.e. EcTBS) and two different stimulation intensities (i.e. at sensory threshold and 110% H-wave threshold). Furthermore, we added another experimental session in which we used a non-patterned stimulation, in which the same number of stimuli was delivered (i.e. 15Hz), to control for a possible pattern-dependency of the after-effects. As both EcTBS and 15Hz stimulation are painful when delivered at 110% of the H-wave reflex, the 15Hz control condition allowed us also to evaluate a possible after-effect induced by pain (independent from the stimulation pattern).

## Methods

### Subjects

A total of 26 healthy subjects without history of neurological disorders were enrolled (9 males, 17 females; mean age ± SD: 32.9 ± 8.8 years; age range: 20–46 years). Three different stimulation protocols were evaluated. Four subjects participated in more than one experimental protocol, which were carried out at least one week apart. One subject did not tolerate the procedure and was excluded from the study. A total of 30 experimental sessions were carried out. The study was performed according to the Declaration of Helsinki and approved by the local Ethics Committee (CEIC-Toledo, Spain). The healthy subjects were informed about the aim of the study and gave their verbal informed consent. The experimental sessions were carried out at *Hospital Nacional de Parapléjicos*, Toledo (Spain).

We did not perform a previous power analysis to calculate sample size because of the explorative nature of the present study.

### Experimental design

**Fig 1:**
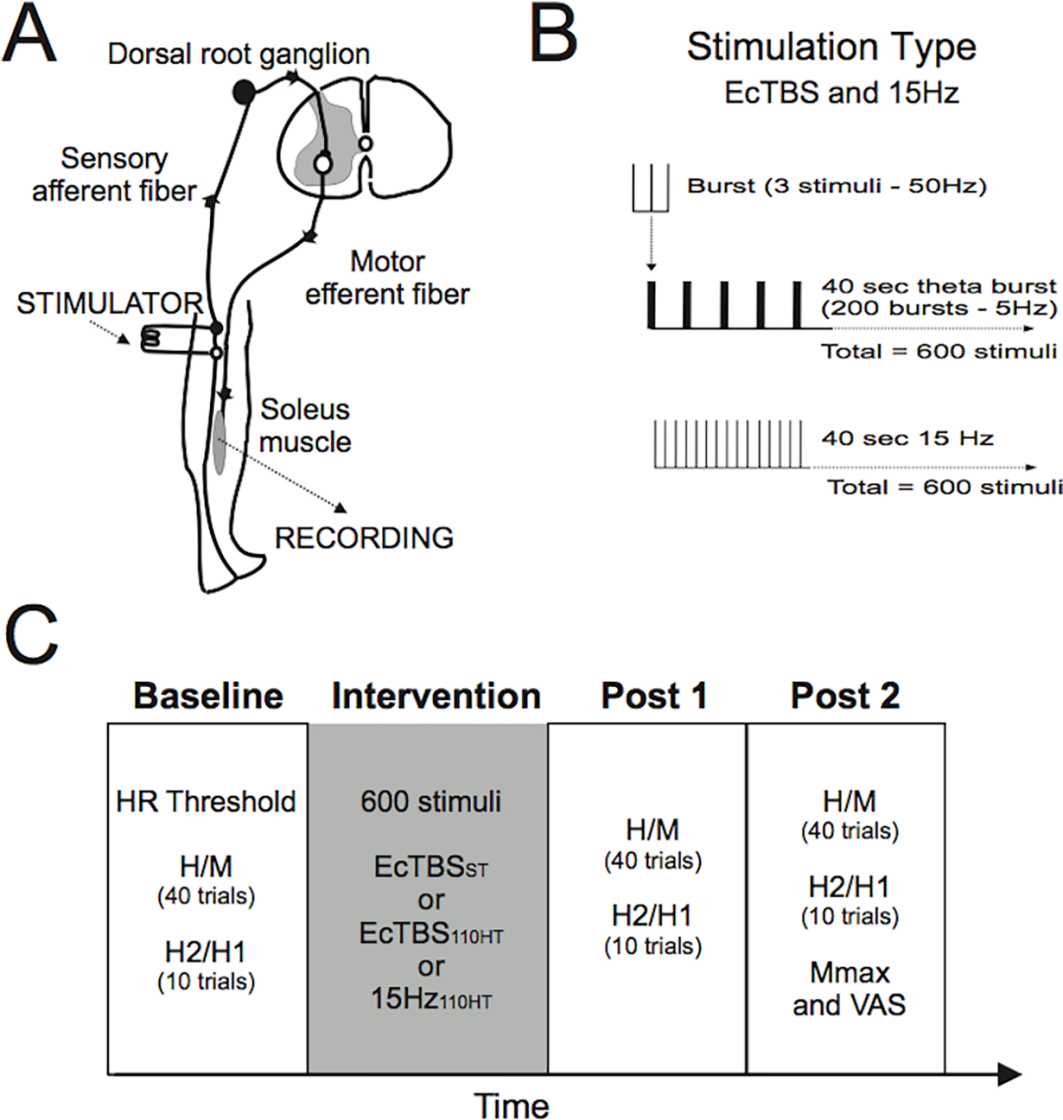
Experimental set-up. A) Schematic view of the monosynaptic reflex and H reflex recordings. B) Theta burst and non-patterned 15Hz interventions. C) The experimental protocol.

We evaluated the effects of peripheral nerve electrical stimulation on spinal reflex excitability of lower limb muscles. We measured the effects on normalized H-reflex amplitude and on paired-pulse inhibition (PPI) of the H-reflex on the soleus muscle. Experimental set-up is summarized in Fig 1.

### Recordings procedures

Each subject was evaluated in a comfortable prone position. Surface electromyography (EMG) was recorded from the right soleus muscle by using adhesive electrodes (Ambu Blue Sensor SnapTab). The active electrode was located over the muscle belly and the reference electrode was located over the Achilles tendon (ground was attached over the proximal portion of the peroneal bone). The EMG was amplified (gain x1000) and band-pass filtered (1Hz to 3 kHz) by Digitimer D360 amplifiers (Digitimer Ltd., Welwyn Garden City, Herts, UK). Signals were recorded at a sampling rate of 10 kHz and stored on the computer for later analysis by Signal software (Cambridge Electronic Design Ltd., Cambridge, UK) through a power 1401 data acquisition interface (Cambridge Electronic Design Ltd., Cambridge, UK). All assessments were performed with the muscle at rest. The EMG background was continuously monitored and traces showing EMG activity were discarded online or offline. To obtain the H reflex, we used a constant current stimulator (DS7A, Digitimer, Welwyn, UK) to apply stimuli of 1 ms pulse width to the tibial nerve. For tibial nerve stimulation, the active electrode was placed in the popliteal fossa (cathodal current) and reference electrode was attached on the skin over the patella through adhesive electrodes (Ambu Blue Sensor SnapTab).

#### Baseline assessments

We assessed baseline reflex excitability by evaluating 1) the H-reflex threshold (HT), 2) the H-reflex amplitude (H), 3) M wave amplitude (M), 4) Maximal M wave (Mmax) and 5) paired-pulse inhibition (PPI) of the H-reflex.

HT was defined as the minimum stimulus current required to evoke H reflexes of amplitude of approximately 50 μV in 50% of trials. To obtain this, stimulus intensities (1–20 mA) were increased in steps of 2 mA until the maximal H-reflex was identified, and then the stimulus intensity was decreased in steps of 1 mA until the H reflex threshold was achieved. Stimuli were delivered every 6–8 s. We used HT only to set the intervention intensity (e.g. HT was not measured again after the intervention).

Afterwards, the stimulation intensity was increased in steps of 0.1–0.5mA until a stable H-reflex with a measurable M wave was obtained. In line with McNulty and coworkers (2008), we chose to record H reflex when a liminal M wave was obtained. This corresponds approximately to an H reflex with amplitude of 70% of Hmax. The advantage of this approach is that H reflex is not saturated and could be increased or decreased in amplitude by a neuromodulation protocol [25]. We used this intensity throughout the entire baseline recording protocol; in which single pulse (20 trials) and paired pulses with two ISIs (50ms and 100ms; 10 trials for each ISI) were randomly delivered. Testing was done at a stimulation rate of 0.2 Hz, as the depressive effects are sufficiently small at this point to justify a rate of one stimulus every 5 s [26]. We utilized paired pulse stimulation (a way to test post-activation depression at spinal level) to compare the recovery profiles of the H-reflex over intervals showing strong inhibition [27]: since strongest inhibition was shown at ISIs between 25 and 250 ms, we arbitrary chose 50 and 100 ms to ensure a shorter and more confortable session for the subjects [28].

In the paired pulse trials, we used the same intensity for the two stimuli. The M-wave and H-wave amplitudes evoked by the single pulse (20 trials) and by the first (unconditioned) of the twenty paired-pulse stimuli were obtained (20 trials). In paired-pulse trials, we will refer as H1 to the H evoked by the first stimulus, and as H2 to the H evoked by the second one. At the end of the whole evaluation, Mmax was recorded by using supramaximal electrical stimulation, and used to normalize H reflex amplitude. Mmax was determined by increasing the intensity of stimulation until the amplitude did not increase anymore. We calculated the H/Mmax ratio as a marker of spinal reflex excitability. The H2/H1 ratio was used to quantify the PPI. The amplitude of the conditioned responses at the two inhibitory ISIs (50 and 100ms) was averaged to give a grand mean value of PPI. The grand mean of the two ISIs has been calculated to reduce the intra-subject variability. The paired pulse study was conducted in 10 subjects with EcTBS_110HT_, 8 subjects with EcTBS_ST_ and 8 with 15Hz_110HT_.

#### Post-intervention assessments

After the intervention, we reassessed the spinal excitability immediately (approximately 1 min after the end of the intervention) and 15 min after the end of the intervention. The two post-intervention assessments were similar to the baseline assessments with two main differences: 1) HT was not re-evaluated and, 2) We looked for an M wave of the exact same amplitude as in the baseline by slightly reducing or increasing the intensity of stimulation. With this procedure, we made sure that the stimuli applied activated a similar number of motor axons in all experimental conditions [29, 30].

### Intervention by patterned or regular electrical peripheral nerve stimulation

We used two peripheral nerve repetitive electrical stimulation protocols: patterned and regular. For patterned stimulation, we applied electrical continuous TBS (EcTBS) consisting of a 3-pulse burst of 50Hz electric stimulation (1ms pulse width) given every 200 ms to the tibial nerve at the popliteal fossa (through the same electrodes used for eliciting H reflex). For regular, non-patterned, electrical stimulation, we applied 15 Hz continuous electrical stimulation (E15S), which was used as control to rule out possible influence of stimulus-induced pain or other factors unrelated to the conditioning stimulation protocol.

For EcTBS, we used two different stimulation intensities: a) 110% of H-reflex threshold and b) sensory threshold intensity. Sensory threshold was determined as the intensity that subjects were able to detect 50 percent of the time (through the same electrodes used for eliciting H reflex and with the same pulse width).

We used 600 pulses, lasting a total of 40 s in all three experimental conditions: 1. EcTBS at 110% H-Reflex threshold intensity (EcTBS_110HT_). 2. EcTBS at 100% of the electrical sensory threshold (EcTBS_ST_), and 3. continuous electrical stimulation at 15Hz at 110% of the H-Reflex threshold intensity (15Hz_110HT_). We evaluated stimulus-induced pain sensation at the end of each experimental session by using a visual analogic scale (VAS), which related to a score of 0 = no pain and 10 = maximum pain.

## Data analysis

Data are reported as mean and standard errors of the mean. Main outcome measures were: 1) H reflex amplitude normalized to the Mmax (H/Mmax); 2) the grand mean of H2/H1 ratio at ISIs of 50ms and 100ms (mean of H2/H1_50ms_ and H2/H1_100ms_) and 3) pain (caused by the interventions at 110%HT). The score obtained from VAS evaluation of stimulus-induced pain was compared among protocols using an unpaired t test. A mixed factorial ANOVA on values normalized to the baseline with factors of TIME (post1/baseline, and post2/baseline) and PROTOCOL (the three GROUPS of subjects submitted to different stimulation conditions: EcTBS_110HT_ and EcTBS_ST_ and 15Hz_110HT_) were used to compare the effects on the H/Mmax ratio between the three different stimulation protocols. TIME was considered as within-subjects factor and PROTOCOL/GROUP was considered as between-subjects factor. Post hoc tests were performed using Tukey Honest. A separate mixed factorial ANOVA on values normalized to the baseline with factors of TIME (post1/baseline, and post2/baseline) and PROTOCOL (the three GROUPS of subjects receiving different intervention: EcTBS_110HT_ and EcTBS_ST_ and 15Hz_110HT_) were used to compare the effects on the H2/H1 ratio between the three different stimulation protocols. Again, TIME was considered as within-subjects factor and PROTOCOL/GROUP was considered as between-subjects factor. Post hoc tests were performed using Tuckey Honest. Differences were considered significant when p<0.05.

For the effect induced by EcTBS_110HT_ on the normalized H reflex amplitude we also calculated the effect size by means of Cohen’s D.

## Results

**Fig 2:**
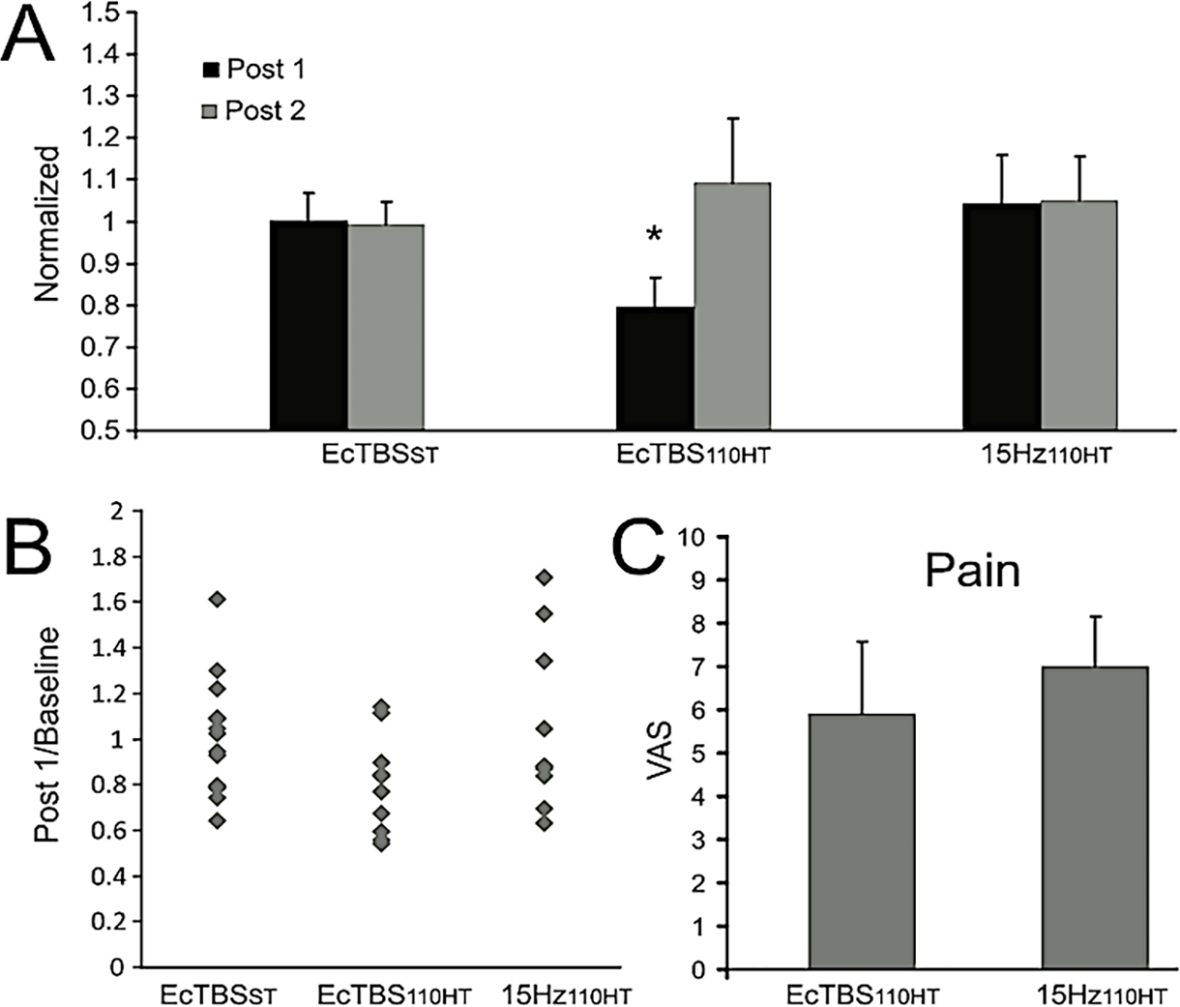
EcTBS_110HT_ main effects. A) Time course of the H/Mmax ratio normalized to the baseline. Values are mean and error bars are standard error of the mean. B) Effects of EcTBS_110HT_ intervention on the H/Mmax ratio in individual subjects (Post 1/Baseline). C) Vas scores in the experiments at above HR thresholds. Vas scores in the experiment at above HR thresholds. Values are mean and error bars are standard deviations.

The subjects that participated in the EcTBS_ST_ reported no pain during the protocol. The subjects, that participated in the experiments at 110% HT reported pain sensation to stimulation trains that did not significantly differ between both, EcTBS_110HT_ and 15Hz_110HT_: (5.9±1.7 and 7±1.2, respectively; p = 0.1029) (Fig 2, panel C). Baseline H-reflex thresholds (EcTBS_110HT_ vs 15Hz_110HT,_ p = 0.268), baseline H-reflex amplitude (EcTBS_110HT_ vs 15Hz_110HT,_ p = 0.757; EcTBS_110HT_ vs EcTBS_ST,_ p = 0.327; 15Hz_110HT_ vs EcTBS_ST,_ p = 0.124), baseline M wave amplitude (EcTBS_110HT_ vs 15Hz_110HT,_ p = 0.367; EcTBS_110HT_ vs EcTBS_ST,_ p = 0.434; 15Hz_110HT_ vs EcTBS_ST,_ p = 0.849) and baseline Mmax amplitude (EcTBS_110HT_ vs 15Hz_110HT,_ p = 0.136; EcTBS_110HT_ vs EcTBS_ST,_ p = 0.554; 15Hz_110HT_ vs EcTBS_ST,_ p = 0.434) were similar in the three protocols (Table 1). Tibial nerve stimulation intensities to obtain a stable H reflex and M wave, in baseline, are reported in Table 1. The intensity used was around 150% HT in EcTBS_110HT_ and 15Hz_110HT_.

**Table 1:** Baseline values for M wave and H reflex evaluation in the three experimental groups: Values are expressed by means ± SD.

	EcTBS_110HT_	EcTBS_ST_	15Hz_110HT_
N	10	10	10
	**Intervention**		
Sensory threshold (mA)		1.2±1.1	
H Reflex threshold (mA)	10.6±4.4		12.8±4.2
Stim. intensity	110% HR threshold	Sensory threshold	110% HR threshold
	**H Reflex Recordings**		
H Reflex (mV)	4.2±2.5	5.2 ± 1.9	3.9±1.7
M Wave (mV)	2.1±1.1	2.7±2.1	2.9±2.5
Stim intensity (mA)	15.8±6.4	16.0 ± 5.7	19.0±6.5
	**Mmax Recordings**		
Mmax (mV)	20.4±5.4	18.7± 7.1	16.3±6.3
Stim Intensity (mA)	28.2±5.6	27.0± 3.9	27.2±4.9

**Fig 3:**
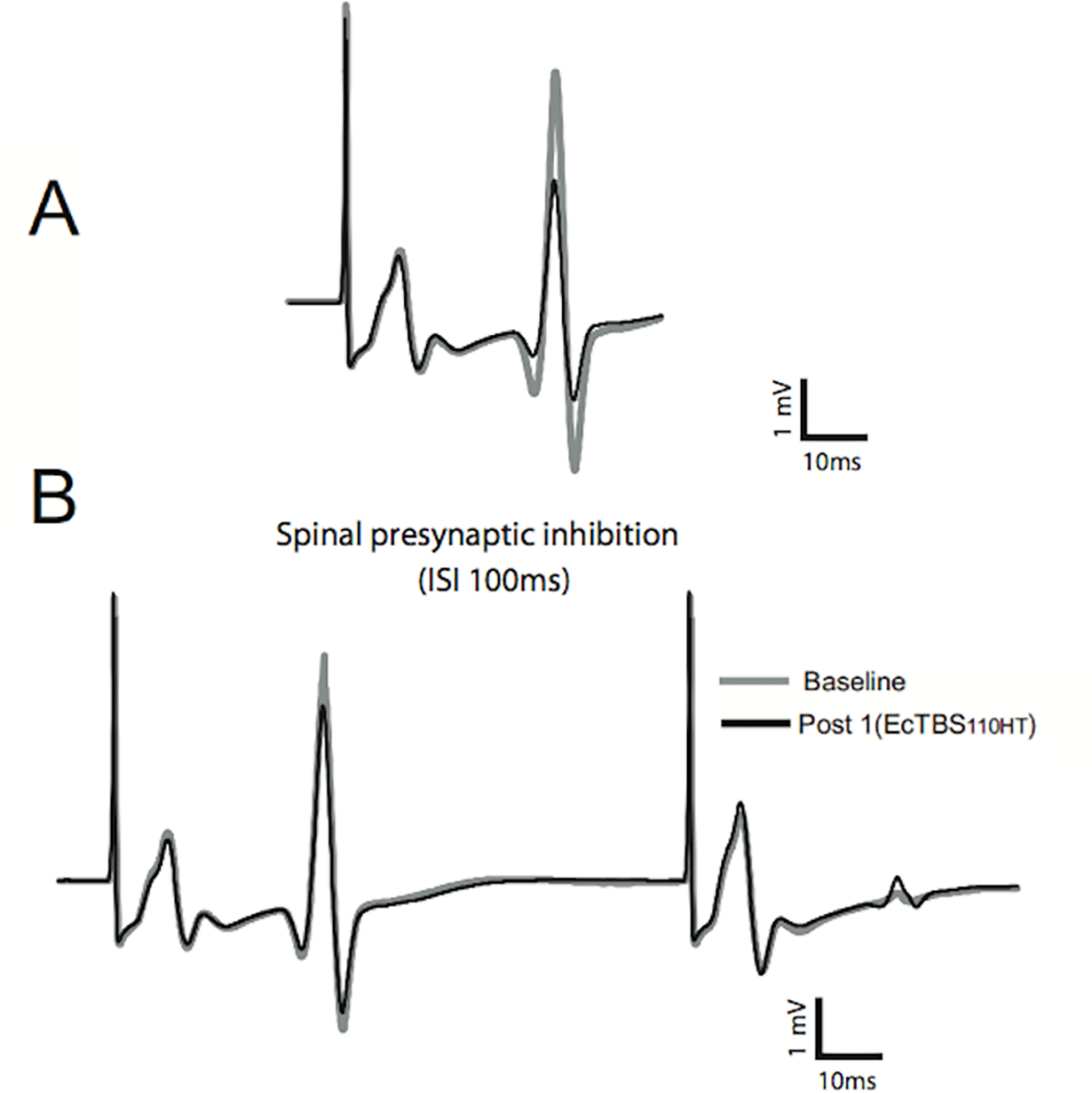
EcTBS_110HT_ effects in a representative subject. A) H reflex in a representative subject before and after EcTBS110HT intervention. B) Paired pulse study (H2/H1) in a representative subject before and after EcTBS110HT intervention.

Fig 3 shows an example of the effects of EcTBS_110HT_ on the H reflex size, with no concomitant effects on the M wave. This was common to all subjects examined in this condition but not in the two other conditions (Fig 2). The statistical analysis showed a significant reduction of the H/Mmax ratio over time depending on the stimulation protocol (ANOVA, TIME x STIM: F_(2,27)_ = 3.340; p = 0.048). The reduction of the H reflex amplitude (and of the H/Mmax ratio) was specific for the EcTBS_110HT_ condition and only for the evaluation carried out just after the end of the neuromodulation protocol (post-hoc t-test, p = 0.047) (Fig 2, panel A and B). Effect size calculation for the first time point (1 minute after the end of EcTBS_110HT_) revealed a medium-large effect induced by EcTBS_110HT_ (d = 0.64).

For PPI of the H-reflex, we did not find any significant effects of the different intervention on the H2/H1 ratio (TIME: F_(1,23)_ = 1.843; p = 0.188; TIME x PROTOCOL: F_(2,23)_ = 0.046; p = 0.955).

## Discussion

In the present study, we show that the application of 40 seconds EcTBS (600 pulses) at 110% of the H-reflex threshold intensity induced a short term reduction of the H/Mmax ratio after the end of the stimulation while EcTBS at ST and 15 Hz non-patterned stimulation did not. None of the stimulation protocols used was able to induce any change in PPI of the H-reflex.

Our first mechanistic consideration is that only the patterned stimulation with an intensity above H threshold (EcTBS_110HT_) was able to affect the excitability of monosynaptic spinal reflex since both the same patterned stimulation below H threshold (i.e. at sensory threshold) and the uniform stimulation (same number of stimuli, same intensity and same main frequency) produced no consistent after effects.

Throughout all the experimental sessions, we kept M wave amplitude constant to assure that peripheral nerve was stimulated at the same efficiency along the experiments. This ensured that the effects observed over H wave amplitude could not be due to changes in M wave amplitude.

Similarly to our experiment, Yeh et al. applied an EcTBS intervention but over the median nerve. They observed that EcTBS at 90% HT was able to reduce the H/M ratio. On the other hand, when they increased the intensity of the peripheral nerve stimulation to 110% H-reflex threshold, the reduction of the H/M ratio was turned into facilitation. They concluded that EcTBS modulates the H-reflex likely through a mechanism involving synaptic plasticity, and that this mechanism depends on the stimulus intensity. They suggest also that stimulation intensity is the key factor to facilitate or suppress the H reflex, arguing that LTD-like phenomena (H/M suppression) were obtained using intensities below H reflex threshold [21]. Conversely, we obtained H reflex amplitude reduction using stimulation intensities above the H reflex threshold. The different sensibility to stimulation intensity of the two studies could be explained by the fact that we studied the lower limbs, while Yeh et al. evaluated the arms. Among many relevant differences, it is worth considering that lower limb muscles are involved in gait, usually characterized by symmetric, rhythmic and gross movements while those of the upper limbs are mainly used in asymmetric and fine movements. Indeed, it was demonstrated that forelimbs and hindlimbs in rats have a different control by descending and/or propriospinal pathways [31] and, similarly, corticofugal descending projections are less prominent for proximal arm or lower limb muscles than for distal arm muscles in humans [32,33]. Hence, it was also demonstrated that leg muscles and arm muscles receive an opposite cortical control on inhibitory spinal networks: leg muscles receive a phasic inhibitory control whereas arm muscle a tonic facilitatory control [34], On the other hand, it should be considered that the organization of lumbar and cervical pathways presents many differences. For example, the lumbar enlargement is characterized by a dominant peripheral facilitation of lumbar premotor neurons whereas in the cervical segment peripheral inhibition prevails [34]. So that, also considering that PPI was not affected and that neuromodulation at sensory threshold was not able to change any studied parameters, it could be hypothesized that tibial nerve EcTBS could have induced plastic phenomena on the spinal motorneuron at post-synaptic level or on the lumbar premotor neurons, reducing their facilitating activity.

Theoretically, H reflex amplitude reduction induced by EcTBS at 110% HT could be caused by other mechanisms, including a change in presynaptic inhibition or in reciprocal inhibition (i.e. increase) at spinal level. It was demonstrated that presynaptic inhibition and post-synaptic depression are both reduced in spasticity and both increased in spinal muscular atrophy patients leading to an opposite change in H reflex amplitude: in few words pathological conditions affecting spinal cord change presynaptic inhibition and post-synaptic depression in the same direction [35]. Our data showed a reduction in H/M ratio after EcTBS at 110% HT without any change in PPI protocol, that represents the gross neurophysiological hallmark of post-synaptic depression: in this sense, although it could not be excluded an effect onto spinal networks controlling presynaptic inhibition and post-synaptic depression, the lack of PPI modulation renders this possibility unlikely.

At the same time we cannot exclude that the activation of cutaneous nerve fibres could have affected our results. Indeed it was shown that patterned stimulation paradigms, by activating that kind of fibres, change reciprocal inhibition at spinal level [36]. Unfortunately we did not assess reciprocal inhibition, but our data unlikely could be explained by a change in reciprocal inhibition. Indeed, the temporal pattern of stimulation was critical: to be effective in changing reciprocal inhibition the patterned stimulation should have pulse trains of more than 100 Hz in frequency because the firing rate of Ia afferents of ankle dorsiflexor muscles increases up to 100–200 Hz during the swing phase of locomotion [36].

EcTBS at sensory threshold was not efficient. This negative result could be explained by the low stimulation intensity used, since H reflex amplitude was modulated by an intensity of 90% and 110% HT [21] and sensory threshold is well below the HT. Furthermore, it was previously shown that a patterned electrical stimulation at motor threshold intensity was able to increase reciprocal inhibition recorded at the soleus muscle [12]. It seems, therefore, that after-effects of conditioning stimulation are highly intensity-dependent. An intensity adjusted at sensory threshold may not be optimal for modulation of H reflex.

Pain may influence the motor system such as the Ia afferent fibre and alpha motoneuron pathway of the H-reflex [37–39]. Peripheral nerve repetitive electrical stimulation in our experiments with intensity above H reflex threshold (110% of H reflex thresholds) was painful. Indeed, pain could have modulated the reflex response to the la afferents. Indeed, we found that non-painful EcTBS_ST_ was not able to modulate H reflex amplitude, while painful EcTBS_110HT_ induced a significant change. However, although it is not possible to completely rule out the influence of pain on H reflex modulation, the fact that the 15Hz_110HT_ stimulation led to a pain score similar to that induced by EcTBS_110HT_ but was not able to induce any modulation in H reflex amplitude, it could be speculated that our findings are specific on the EcTBS_110HT_ protocol.

None of the interventional protocols applied in this study had effects on the PPI of the H-reflex that is an index of post-activation or homosynaptic depression. PPI refers to a reduction of H reflex amplitude in response to a previously activated sensory-motor synapse, associated with a change in the Ia afferent terminal leading to a transient reduction in neurotransmitter release at pre-synaptic level [30,40–42]. On the other hand, the size of H reflex depends on the synaptic activation of motorneurons by group Ia muscle afferents [5]. Considering that ECTBS_110HT_ did not change PPI and that pain induction was not crucial in the H reflex modulation, it could be hypothesized that EcTBS_110HT_ does not directly affect the excitability of Ia fibres or the efficiency of the synapses between Ia fibres and motorneurons but probably produces a change in spinal motorneurons’ excitability. Again, since the only protocol able to change H-reflex amplitude was ECTBS_110HT_, it could be speculated that a specific stimulation pattern is required to “activate” this adaptation. Previously it was demonstrated that only patterned peripheral electrical stimulation protocols could modulate reciprocal inhibition, another form of spinal inhibition that share some mechanisms with post-activation or homosynaptic depression [12]. In this respect, our findings seem in contrast with those of Perez and collaborators. However it should be noted that our protocols used a small number of stimuli (i.e. 600 stimuli) whereas a total number of 10000 stimuli were used by the other group: this methodological discrepancy could have accounted for our negative findings, since it was shown that inhibition prevails on the long run [13].

Furthermore, we did not perform additional experiments in which cTBS was applied at different intensities or with longer duration to study in more details the influence of these variables on the after-effects of peripheral cTBS.

We conceived this study as a proof of principle study so we concentrated only on H reflex and post-activation or homosynaptic depression without evaluating other spinal phenomena, such as reciprocal inhibition, non-reciprocal inhibition, recurrent inhibition and so on [5]. It should be considered that our results were confined to a modulation of the H-reflex circuitry as indexed by a reduction in H/Mmax. A re-evaluation of the H threshold after intervention might have added more information about the nature of modulation. On the other hand, we did not re-test H threshold to avoid delaying the evaluation of H wave amplitude changes. It should be noted that the effects of EcTBS on H/Mmax were transient, as they were only present at 1 min but not at 15 min after the end of EcTBS. Therefore, our preliminary results should be considered with caution. Further studies are warranted to give more mechanistic insights into EcTBS effects on spinal excitability. It needs to be clarified whether the effects of EcTBS can be prolonged by increasing the duration of the intervention. The ability to induce longer lasting effects on spinal excitability is a prerequisite for clinical application of the technique.

In conclusion, we showed that TBS patterned electrical stimulation, even in the presence of a certain interindividual variability, is efficient to modify spinal excitability also in lower limb circuitries. Hitherto, spinal paired associative stimulation and tsDCS require a relatively lengthy stimulation to produce effects while TBS has the advantage to be very much shorter than those. Further studies on spinal plasticity induced by peripheral nerve stimulation using EcTBS would be important for a better understanding of human spinal cord physiology.

## Supporting information

S1 File Experimental protocol. Details about participants, neurophysiological evaluations, interventions and data analysis are provided.
(DOCX)

S2 File Data set. (XLSX)
