# Supplementary material for: Effects of patterned peripheral nerve stimulation on soleus spinal motor neuron excitability
Source: PLoS One. 2018 Feb 16;13(2):e0192471. doi: 10.1371/journal.pone.0192471 (PMC5815584; doi:10.1371/journal.pone.0192471)
Supplement: S1 File — Details about participants, neurophysiological evaluations, interventions and data analysis are provided. (DOCX) [file pone.0192471.s001.docx]

PROTOCOLO EXPERIMENTAL

Se evaluarán los efectos de la estimulación eléctrica repetitiva de un nervio periférico sobre la excitabilidad de los reflejos espinales en miembros inferiores. Para ello se medirá sobre el músculo sóleo los efectos de esta estimulación sobre la amplitud del reflejo H, así como los efectos de la inhibición inducida por pulsos pareados.

**Sujetos de estudio**

Se reclutarán sujetos sanos sin historia de enfermedades neurológicas con un rango de edad comprendido entre los 25 y los 50 años.

El tamaño de la muestra calculado para el presente estudio es de 20 personas basándonos en las muestras utilizadas por estudios similares.

**Procedimiento de registro**

Para el estudio los sujetos serán colocados en posición decúbito prono. A continuación, se registrará la electromiografía de superficie sobre el músculo sóleo derecho mediante electrodos adhesivos utilizando un montaje donde el electrodo activo se coloca sobre el vientre del músculo y el de referencia sobre la inserción del tendón. La señal electromiográfica será amplificada y filtrada mediante un filtro pasa banda (1Hz-3 kHz), todo ello con un equipo de amplificadores Digitimer D360 (Digitimer Ltd., Welwyn Garden City, Herts, UK). La señal se obtendrá mediante un conversor analógico digital 1401 (Cambridge Electronic Design Ltd., Cambridge, UK) con una frecuencia de muestreo de 10 kHz y se almacenará en un ordenador portátil para su posterior análisis mediante el software Signal (Cambridge Electronic Design Ltd., Cambridge, UK.

La onda H se evocará mediante un estimulador eléctrico de corriente continua (DS7A, Digitimer, Welwyn, UK) que aplicará pulsos de 1ms de duración sobre el nervio tibial posterior a nivel de la fosa poplítea.

*Valoración basal*

El reflejo H en condición basal se evaluará de la siguiente manera:

1) Umbral de la onda H

2) Amplitud de la onda H

3) Amplitud de la onda M

4) Onda M máxima

5) Inhibición de la onda H mediante pulsos pareados.

El umbral del reflejo H será definido como la intensidad mínima de estímulo necesaria para evocar el mencionado reflejo con una amplitud de respuesta de aproximadamente 50 µV en el 50% de las respuestas.

Esta intensidad sólo será utilizada para fijar la intensidad durante la intervención.

Para registrar la onda H, la intensidad de estimulación se obtendrá aumentando la intensidad de estimulación en pasos de 0.1 a 0.5mA hasta obtener una repuesta estable acompañada de una onda M fácilmente medible.

Esta intensidad se utilizará durante todo el proceso de registro en condición basal donde se aplicarán 20 pulsos simples y 20 pulsos pareados (10 con 50ms y 10 con 100 ms de intervalo), todo ellos aplicados de manera randomizada.

La frecuencia utilizada de registro será de 0.2 Hz.

En el caso de los estímulos pareados, se utilizará la misma intensidad para los dos pulsos.

Se medirán las amplitudes tanto de la onda M como de la H evocadas por los pulsos simples y por los pareados.

Al final de la sesión, la onda M máxima será registrada mediante estimulación eléctrica supramaximal y ésta se utilizará para normalizar la amplitud de la onda H.

La M máxima se obtendrá aumentando la intensidad de estimulación ahasta que la amplitude de la onda M no se incremente más.

La ratio H/Mmáx será considerada como marcador de la excitabilidad de los reflejos espinales.

La ratio H2/H1 será considerada como marcador de la inhibición por pulsos pareados.

La amplitud de las respuestas condicionadas por los dos tipos de intervalo interestímulo (50 y 100ms) serán promediadas para obtener un valor promedio general de la inhibición por pulsos pareados.

*Valoración postintervención*

Tras la intervención, se reevaluará la excitabilidad espinal inmediatamente después y tras 15 minutos desde el fin de la intervención.

Las dos valoraciones postintervención serán iguales a las realizadas durante la valoración basal excepto por dos aspectos: El umbral del reflejo H no será evaluado y la onda H será registrada con la misma amplitud que la obtenida en condición basal aumentando o disminuyendo para ello la intensidad de la estimulación eléctrica aplicada. De esta manera se asegura que la estimulación aplicada activa un número similar de axones motores a lo largo de todas las condiciones experimentales.

**Intervención**

*Estimulación eléctrica de nervio periférico con patrón o regular*

Se aplicarán dos protocolos de estimulación eléctrica repetitiva de nervio periférico: con patrón o regular.

Para la estimulación con patrón, se aplicará estimulación eléctrica continua thetaburst (EcTBS) que consiste en la aplicación de 3 pulsos a 50Hz (burst) repetidos cada 200ms sobre el músculo tibial posterior a nivel de la fosa poplítea (mediante los mismos electrodos utilizados para evocar el reflejo H).

Para la estimulación eléctrica regular o sin patrón, se aplicará estimulación eléctrica a una frecuencia continua de 15 Hz. Ésta se utilizará como control para descartar una posible influencia del dolor derivado del pulso de estimulación u otros factores no relacionados con el protocolo de estimulación.

Intensidades de estimulación:

-110% del umbral de la onda H

-Umbral sensitivo: será definido como la mínima intensidad de estímulo percibida por el sujeto en el 50 % de las veces.

Se aplicarán un total de 600 pulsos que tendrán una duración de 40s en las tres condiciones experimentales:

1. EcTBS al 110% del umbral de la onda H.

2. EcTBS al 100% del umbral sensitivo.

3. Estimulación eléctrica contínua a 15Hz al 110% del umbral de la onda H.

El dolor inducido por el pulso de estimulación será evaluado en cada sesión experimental mediante la escala visual analógica (EVA) cuya puntuación va de 0 a 10 siendo 0 nada de dolor y 10 el máximo dolor perceptible.

**Análisis de los datos**

Datos a valorar:

1) Amplitud de la onda H normalizada a la M máx.

2) La media general de la ratio H2/H1 con los intervalos interestímulo a 50ms y 100ms.

3) Dolor (causado por las intervenciones aplicadas al 110% del umbral de la onda H).

Las puntuaciones obtenidas de la evaluación mediante la escala EVA para cada uno de los protocolos experimentales serán comparadas mediante la prueba t de Student desapareada.

Se utilizará una ANOVA factorial mixta con valores normalizados a la condición basal para comparar los efectos inducidos sobre la ratio H/Mmáx entre los tres diferentes protocolos de estimulación. Se utilizarán los factores TIEMPO (post1/basal y post2/basa) y PROTOCOLO.

El TIEMPO será considerado como factor intra-sujetos y PROTOCOLO/GRUPO como factor inter-sujetos.

Se realizará una segunda ANOVA factorial mixta con valores normalizados a la condición basal y con los factores TIEMPO (post1/basal, y post2/basal) y PROTOCOLO, para comparar los efectos sobre la ratio H2/H1 entre los tres diferentes protocolos de estimulación.

De nuevo, el factor TIEMPO será considerado como factor intra-sujetos y PROTOCOLO/GRUPO como factor inter-sujetos.

Las diferencias serán consideradas significativas cuando los valores de p sean p<0.05.

EXPERIMENTAL PROTOCOL

TRANSLATED FROM SPANISH ORIGINAL PROTOCOL

The effects of peripheral nerve electrical stimulation on spinal reflex excitability of lower limb muscle will be evaluated. For this, we will measure the effects on H-reflex amplitude and on paired-pulse inhibition of the H-reflex on the soleus muscle.

**Participants**

Healthy subjects without history of neurological disorders will be recruited, age range 25-50 years.

We estimate a sample size of 20 subject as those used in similar previous studies.

**Recordings procedures**

Subjects will be evaluated in a comfortable prone position. Surface electromyography (EMG) will be recorded from the right soleus muscle by using adhesive electrodes in a belly tendon montage. The EMG will be amplified and band-pass filtered (1Hz to 3 kHz) by Digitimer D360 amplifiers (Digitimer Ltd., Welwyn Garden City, Herts, UK). Signals will be recorded at a sampling rate of 10 kHz and stored on the computer for later analysis by Signal software (Cambridge Electronic Design Ltd., Cambridge, UK) through a power 1401 data acquisition interface (Cambridge Electronic Design Ltd., Cambridge, UK).

To obtain the H reflex, we will use a constant current stimulator (DS7A, Digitimer, Welwyn, UK) by applying stimuli of 1 ms pulse width to the tibial nerve in the popliteal fossa.

*Baseline assessments*

Baseline H-reflex excitability will be assessed by evaluating:

1) H-reflex threshold

2) H-reflex amplitude

3) M wave amplitude

4) Maximal M wave

5) Paired-pulse inhibition of the H-reflex.

H-reflex threshold will be defined as the minimum stimulus current required to evoke H reflexes of amplitude of approximately 50 µV in 50% of trials.

H-reflex threshold intensity will be used only to set the intervention intensity.

For H-reflex recording, the stimulation intensity will be increased in steps of 0.1-0.5mA until a stable H-reflex with a measurable M wave will be obtained.

This intensity will be used throughout the entire baseline recording protocol; in which single pulse (20 trials) and paired pulses with two ISIs (50ms and 100ms; 10 trials for each ISI) will be randomly delivered.

Testing will be done at a stimulation rate of 0.2 Hz.

In the paired pulse trials, we will use the same intensity for the two stimuli.

The M-wave and H-reflex amplitudes evoked by the single pulse (20 trials) and by the first of the twenty paired-pulse stimuli will be obtained.

At the end of the whole evaluation, Maximal M wave will be recorded by using supramaximal electrical stimulation, and used to normalize H reflex amplitude.

Maximal M wave will be obtained increasing the intensity of stimulation until the amplitude do not increase anymore.

The H/Mmax ratio will be calculated as a marker of spinal reflex excitability.

The H2/H1 ratio will be determine to quantify the paired pulse inhibition.

The amplitude of the conditioned responses at the two inhibitory ISIs (50 and 100ms) will be averaged to give a grand mean value of paired pulse inhibition.

*Post-intervention assessments*

After the intervention, the spinal excitability will be reassessed immediately after and 15 min after the end of the intervention.

The two post-intervention assessments will be like the baseline assessments except for: H-reflex threshold won’t be evaluated and an M wave of the exact same amplitude as in the baseline will be determine by slightly reducing or increasing the intensity of stimulation to ensure that the stimuli applied activate a similar number of motor axons in all experimental conditions.

**Intervention**

*Patterned or regular electrical peripheral nerve stimulation*

We will use two peripheral nerve repetitive electrical stimulation protocols: patterned and regular.

For patterned stimulation, we will apply electrical continuous TBS (EcTBS) consisting of a 3-pulse burst of 50Hz electric stimulation (1ms pulse width) given every 200 ms to the tibial nerve at the popliteal fossa (through the same electrodes used for eliciting H reflex).

For regular, non-patterned, electrical stimulation, we will apply 15 Hz continuous electrical stimulation, that will be use as a control to rule out possible influence of stimulus-induced pain or other factors unrelated to the conditioning stimulation protocol.

Stimulation intensities:

-110% of H-reflex threshold

-Sensory threshold intensity: defined as the minimum stimulus current felt by the subject the 50% of times.

A total of 600 pulses will be delivered, lasting a total of 40 s in all three experimental conditions:

1. EcTBS at 110% H-Reflex threshold intensity.

2. EcTBS at 100% of sensory threshold.

3. Continuous electrical stimulation at 15Hz at 110% of the H-Reflex threshold intensity.

Stimulus-induced pain will be evaluated at the end of each experimental session by means of a visual analogic scale (VAS), scored of 0= no pain and 10= maximum pain.

**Data Analysis**

Main outcome measures:

1) H-reflex amplitude normalized to the Mmax

2) The grand mean of H2/H1 ratio at ISIs of 50ms and 100ms

3) Pain (caused by the interventions at 110% H-reflex threshold).

The scores obtained from VAS evaluation of stimulus-induced pain will be compare among protocols using an unpaired t test.

A mixed factorial ANOVA on values normalized to the baseline with factors of TIME (post1/baseline, and post2/baseline) and PROTOCOL will be used to compare the effects on the H/Mmax ratio between the three different stimulation protocols.

TIME will be considered as within-subjects factor and PROTOCOL/GROUP will be considered as between-subjects factor.

Another mixed factorial ANOVA on values normalized to the baseline with factors of TIME (post1/baseline, and post2/baseline) and PROTOCOL will be used to compare the effects on the H2/H1 ratio between the three different stimulation protocols.

Again, TIME will be considered as within-subjects factor and PROTOCOL/GROUP will be considered as between-subjects factor.

Differences will be considered significant when p<0.05.
